# Replication of acne susceptibility loci and gene–environment interactions with screen time in Singapore and Malaysia Chinese population

**DOI:** 10.1016/j.xjidi.2026.100490

**Published:** 2026-05-08

**Authors:** Lingshu Liu, Yang Yie Sio, Dingyu Cen, Jia Yi Karen Wong, Xiangran Chen, Yee-How Say, Kavita Reginald, Fook Tim Chew

**Affiliations:** 1 Department of Biological Sciences, National University of Singapore, Singapore, Singapore; 2 Department of Biomedical Science, Faculty of Science, Universiti Tunku Abdul Rahman (UTAR) Kampar Campus, Kampar, Malaysia; 3 Department of Biomedical Sciences, Sir Jeffrey Cheah Sunway Medical School, Faculty of Medical and Life Sciences, Sunway University, Subang Jaya, Malaysia

**Keywords:** Acne vulgaris, Gene–environment interaction, Genetic susceptibility, Single nucleotide variant, Genome-wide association study

## Abstract

Acne vulgaris is a common chronic inflammatory skin disorder with a substantial genetic contribution. However, replication of findings from genome-wide association studies across diverse populations remains limited. In this study, we evaluated 88 previously reported acne-associated variants in 2741 acne cases and 2235 controls from the Singapore/Malaysia Cross-sequential Genetic Epidemiology Study. Two association signals were replicated at Bonferroni-corrected significance: rs1159268 near *TGFB2* at 1q41 and rs738409 in the *PNPLA3* coding region at 22q13.31, with consistent directions of effect. Functional annotation and transcriptomic evidence supported the involvement of these loci in pathways related to pilosebaceous unit biology, including epithelial differentiation, tissue homeostasis, retinoid regulation, and lipid metabolism. Gene–environment interaction analyses further identified 12 variants whose associations with acne risk were modified by screen-time exposure. These findings suggest that screen-associated exposures may act as contextual modifiers of genetic susceptibility, potentially through lifestyle-related metabolic factors, circadian endocrine regulation, and oxidative stress responses. Together, these findings emphasize the importance of considering environmental exposures alongside genetic susceptibility to refine our understanding of acne pathogenesis.

## Introduction

Acne vulgaris is one of the most prevalent chronic inflammatory skin disorders, primarily affecting the pilosebaceous unit. It affects up to 85% of adolescents and young adults and approximately 9–10% of the global population, constituting a substantial worldwide disease burden (Tan and Bhate, 2015; Williams et al, 2012). Acne pathogenesis is multifactorial, involving increased sebum production, follicular hyperkeratinization, colonization with *Cutibacterium acnes*, and dysregulated inflammatory responses, with hormonal and metabolic factors further modulating disease activity (Tan and Bhate, 2015; Williams et al, 2012).

Acne vulgaris has a strong genetic basis, with heritability estimated at 70–80% (Bataille et al, 2002). Previous systematic reviews suggested a heterogeneous genetic architecture involving inflammatory signaling, androgen regulation, lipid metabolism, and keratinization pathways (Heng et al, 2021). Building on these early investigations, genome-wide association studies (GWASs) have substantially advanced our understanding of the biological basis of acne. Although initial studies in Han Chinese populations identified loci at 1q24.2 and 11p11.2 (He et al, 2014), recent expansive meta-analyses in European-ancestry cohorts have identified approximately 50 susceptibility loci (Mitchell et al, 2022; Teder-Laving et al, 2024). Notably, functional genomic evidence has shifted the pathogenic framework from innate immunity toward hair follicle development, morphogenesis, and tissue remodeling (Mitchell et al, 2022; Petridis et al, 2018). Specifically, key risk genes such as *WNT10A*, *LGR6*, and *TP63* underscore the critical role of pilosebaceous unit structure and homeostasis in establishing a follicular environment prone to comedogenesis (Petridis et al, 2018). Consequently, core signaling pathways, including Wnt and mitogen-activated protein kinase pathways, have emerged as central regulators of genetic susceptibility, whereas lipid biosynthesis pathways may act as downstream contributors (Teder-Laving et al, 2024). However, the relative lack of cross-ancestry replication underscores the need for further validation in underrepresented populations to achieve a more globally representative understanding of acne genetics.

Beyond inherited susceptibility, environmental and lifestyle factors also play an important role in acne risk and severity. Epidemiological studies consistently implicate family history, age, body mass index, and skin type, whereas associations with other exposures, such as diet and smoking, remains heterogeneous across populations (Heng and Chew, 2020). Dietary factors have received particular attention: high glycemic index dietary patterns and dairy intake have been associated with acne, plausibly through insulin-like growth factor-mediated activation of signaling pathways involving phosphoinositide 3-kinase, protein kinase B, FOXO1, and mechanistic target of rapamycin complex 1 in sebocytes and keratinocytes (Burris et al, 2013; Melnik and Zouboulis, 2013). Recent nutrigenomic studies further support this framework by demonstrating that glycemic dietary exposures can interact with genetic variation to modulate acne risk, implicating molecular pathways converging on insulin-like growth factor 1 signaling, FOXO1 activity, and mechanistic target of rapamycin complex 1 beyond simple dietary main effects (Say et al, 2022). Psychological stress and environmental pollutants have also been associated with acne exacerbation in observational and time-series studies (El Haddad et al, 2021; Zari and Alrahmani, 2017). Together, these findings highlight that acne arises from the interplay of genetic predisposition with diverse environmental factors, and many contemporary lifestyle exposures remain insufficiently characterized in relation to acne risk (Heng and Chew, 2020).

Gene–environment interaction (G×E) analysis offers a powerful framework to elucidate how genetic susceptibility is modified by environmental context and may account for disease variance unexplained by main-effect GWAS alone (Hunter, 2005; Manolio et al, 2009). Incorporating environmental exposures into genetic models has revealed biologically meaningful interactions in other complex traits (Ober and Vercelli, 2011; Thomas, 2010), yet G×E studies in acne are scarce, especially in non-European populations. In this study, we aimed (i) to replicate previously reported acne-associated loci in the Singapore/Malaysia Cross-sequential Genetic Epidemiology Study (SMCGES) cohort and (ii) to investigate G×Es in this population, with a particular focus on contemporary lifestyle factors. Through this approach, we seek to gain deeper insight into the combined genetic and environmental determinants of acne risk in Southeast Asia populations.

## Results

### Identification of previously reported acne-associated variants

**Figure 1:**
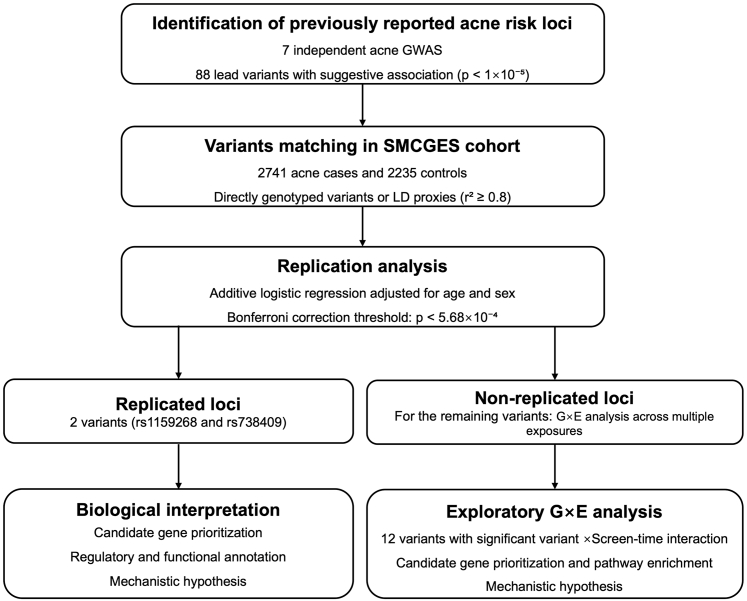
**Study workflow for replication of reported acne loci and G×E analyses.** A total of 88 lead variants (*P* < 1 × 10^−5^) from 7 published acne GWASs were evaluated in the SMCGES cohort. Variants were mapped to directly genotyped variants or proxy variants in high linkage disequilibrium (r^2^ ≥ 0.8). Replication analyses were performed using additive logistic regression adjusted for age and sex, with Bonferroni correction (*P* < 5.68 × 10^−4^). Two loci met the replication threshold and were carried forward for downstream annotation. Non-replicated variants were subsequently evaluated in G×E analyses, identifying 12 variants with significant interaction with screen-time exposure. GWAS, genome-wide association studies; G×E, gene–environment interaction; LD, linkage disequilibrium; SMCGES, Singapore/Malaysia Cross-sequential Genetic Epidemiology Study.

A systematic literature search identified 7 independent GWAS on acne (Figure 1). From these studies, 88 previously reported acne-associated variants were extracted for replication analysis (Supplementary Table S1). All variants had shown suggestive evidence of association in previous reports, defined by a significance threshold of *P* < 1 × 10^−5^. Among them, 5 variants (rs80293268, rs1256580, rs2901000, rs629725, and rs34560261) had been reported in more than 1 previous acne GWAS.

### Cohort characteristics of the SMCGES dataset

**Figure 2:**
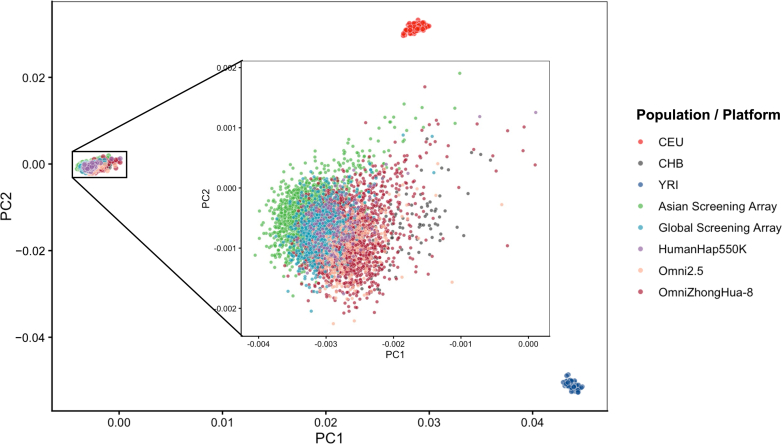
**Population structure of the study cohort relative to reference populations and genotyping platforms.** Principal component analysis plot of the study cohort and reference populations from the 1000 Genomes Project. Each point represents an individual sample plotted according to the first two principal components (PC1 and PC2). The study cohort (n = 4976) clusters closely with the CHB reference population. The inset shows a magnified view of the CHB cluster, with samples colored by genotyping platform. No apparent stratification across platforms is observed. CEU, Utah residents with Northern and Western European ancestry; CHB, Han Chinese in Beijing, China; PC1, first principal component; PC2, second principal component; YRI, Yoruba in Ibadan, Nigeria.

Replication analyses were conducted in the Chinese subset of the SMCGES cohort, comprising 2741 acne cases and 2235 nonacne controls (Table 1). The study population included detailed demographic information and a broad range of environmental and lifestyle exposures. Comparative analyses indicated that age and screen usage time differed significantly between cases and controls, whereas other variables showed broadly comparable distributions. To further characterize the genetic background of the study population, principal component analysis was performed. The cohort clustered closely with the 1000 Genomes Han Chinese in Beijing (CHB) reference population, with no evident stratification across genotyping platforms (Figure 2). Detailed sample characteristics are summarized in Table 1.

**Table 1:** Detailed Demographics of the SMCGES Cohort

Demographics^1^^,^^4^	Total	Acne^2^	Nonacne^2^	*P*-Value^3^
Sample size, n	4976	2741	2235	—
Sex: male, n (%)	2083 (41.86)	1121 (40.90)	962 (43.04)	Reference
Female, n (%)	2870 (57.68)	1607 (58.63)	1263 (56.51)	N.S.
Age, y, mean ± 1 SD	22.75 ± 5.49	22.46 ± 4.65	23.11 ± 6.35	.001∗∗∗
BMI (kg/m^2^), mean ± 1 SD	21.26 ± 3.23	21.25 ± 3.18	21.27 ± 3.29	N.S.
Screen usage time: <1 h/d, n (%)	484 (9.73)	224 (8.17)	260 (11.63)	Reference
Screen usage time: 1–3 h/d, n (%)	1332 (26.77)	704 (25.68)	628 (28.1)	.05∗
Screen usage time: 3–5 h/d, n (%)	1308 (26.29)	746 (27.22)	562 (25.15)	<.001∗∗∗
Screen usage time: >5 h/d, n (%)	1749 (35.15)	1016 (37.07)	733 (32.8)	<.001∗∗∗
Physical activity frequency: never/occasionally, n (%)	2597 (52.19)	1418 (51.73)	1179 (52.75)	Reference
Physical activity frequency: 1–2/wk, n (%)	607 (12.2)	343 (12.51)	264 (11.81)	N.S.
Physical activity frequency: most/all days, n (%)	1675 (33.66)	928 (33.86)	747 (33.42)	N.S.
Housing type: government-built apartment, n (%)	2865 (57.58)	1551 (56.59)	1314 (58.79)	Reference
Housing type: condominium, n (%)	962 (19.33)	514 (18.75)	448 (20.04)	N.S.
Housing type: landed property, n (%)	813 (16.34)	481 (17.55)	332 (14.85)	.05∗
Income: <$2000, n (%)	769 (15.45)	408 (14.89)	361 (16.15)	Reference
Income: $2000–3999, n (%)	1489 (29.92)	840 (30.65)	649 (29.04)	N.S.
Income: $4000–5999, n (%)	1072 (21.54)	594 (21.67)	478 (21.39)	N.S.
Income: >$6000, n (%)	1377 (27.67)	748 (27.29)	629 (28.14)	N.S.
Milk intake: never/occasionally, n (%)	1002 (20.14)	565 (20.61)	437 (19.55)	Reference
Milk intake: once or twice/wk, n (%)	2174 (43.69)	1195 (43.6)	979 (43.8)	N.S.
Milk intake: most/all days, n (%)	1700 (34.16)	926 (33.78)	774 (34.63)	N.S.
Seafood intake: never/occasionally, n (%)	432 (8.68)	246 (8.97)	186 (8.32)	Reference
Seafood intake: once or twice/week (n, %)	2530 (50.84)	1398 (51)	1132 (50.65)	N.S.
Seafood intake: most/all days, n (%)	1921 (38.61)	1043 (38.05)	878 (39.28)	N.S.
Fast food intake: never/occasionally, n (%)	1750 (35.17)	959 (34.99)	791 (35.39)	Reference
Fast food intake: once or twice/wk, n (%)	2749 (55.25)	1527 (55.71)	1222 (54.68)	N.S.
Fast food intake: most/all d, n (%)	373 (7.5)	197 (7.19)	176 (7.87)	N.S.
Alcohol intake: nondrinker, n (%)	120 (2.41)	64 (2.33)	56 (2.51)	Reference
Alcohol intake: occasional, n (%)	2709 (54.44)	1523 (55.56)	1186 (53.06)	N.S.
Alcohol intake: frequent, n (%)	2060 (41.4)	1105 (40.31)	955 (42.73)	N.S.

Abbreviations: BMI, body mass index; N.S., nonsignificant; SMCGES, Singapore/Malaysia Cross-sequential Genetic Epidemiology Study.

1 Continuous variables are presented as mean ± SD; categorical variables are reported as n (%).

2 Acne was defined as a physician-diagnosed history of acne and/or the presence of acne scarring.

3 Fisher’s exact test was used for categorical variables, and independent samples *t*-tests were applied for continuous variables; statistical significance was indicated as ∗*P* < .05, ∗∗*P* < .01, and ∗∗∗*P* < .001; N.S.: *P* > .05.

4 Some varia bles contained missing observations; percentages were calculated on the basis of nonmissing data and may not total 100%.

### Replication analysis of previously reported acne-associated variants

Of the 88 previously reported acne-associated variants, 71 were represented in the SMCGES dataset by either the reported variant itself or a proxy variant in strong linkage disequilibrium (r^2^ ≥ 0.8). After accounting for duplicate proxy mapping, these corresponded to 68 unique variants that were tested in the replication analysis (Supplementary Table S2). Additive logistic regression identified 2 variants that exceeded the conservative study-wide significance threshold on the basis of the 88 literature-derived acne-associated variants after Bonferroni correction (*P* < 5.68 × 10^−4^) (Table 2). These were rs1159268 at 1q41 and rs738409 at 22q13.31. Both variants showed effects in the same direction as previously reported. No additional variants met the corrected significance threshold.

**Table 2:** Replication of Previously Reported Acne-Associated Genetic Variants in the SMCGES Cohort

rsID	Chr:Pos (GRCh37)	Implicated Gene^1^	RA/PA	Reported Study					Replication in SMCGES Cohort^2^			
				Population	N (Ca/Co)	RAF	OR (95% CI)	*P*-Value	N (Ca/Co)	RAF	OR (95% CI)	*P*-Value^3^
rs1159268	1:218844906	*TGFB2*	A/G	UK Population	3956 /7102	0.37	1.17 (1.08–1.27)	4.08E-08	2741/2235	0.51	1.149 (1.063–1.245)	5.53E-04
rs738409	22:44324727	*PNPLA3*	C/G	European	20165/595231	0.78	1.09 (1.06–1.12)	1.00E-08	2741/2235	0.62	1.178 (1.083–1.280)	1.22E-04

Abbreviations: Ca, cases; Chr, chromosome; CI, confidence interval; dbSNP, database of single-nucleotide variant; eQTL, expression quantitative trait locus; Co, controls; GTEx, Genotype-Tissue Expression; ID, identification; N, number of the sample size; PA, protective allele; Pos, position; RA, risk allele; RAF, risk allele frequency; SMCGES, Singapore/Malaysia Cross-sequential Genetic Epidemiology Study; UK, United Kingdom; UTR, untranslated region.

1 Implicated genes were determined by (i) dbSNP functional annotation (coding, splice-site, UTR) to protein-coding genes; (ii) significant eQTLs in acne-relevant GTEx tissues (skin, subcutaneous adipose, cultured fibroblasts, whole blood); and (iii) otherwise, the nearest protein-coding gene.

2 ORs and *P*-values were estimated using a logistic regression model adjusted for age and sex.

3 Replication significance was defined using a Bonferroni-corrected threshold of *P* < .05/88, corresponding to the 88 previously reported variants tested.

### Functional annotation of the replicated variants rs1159268 and rs738409

**Figure 3:**
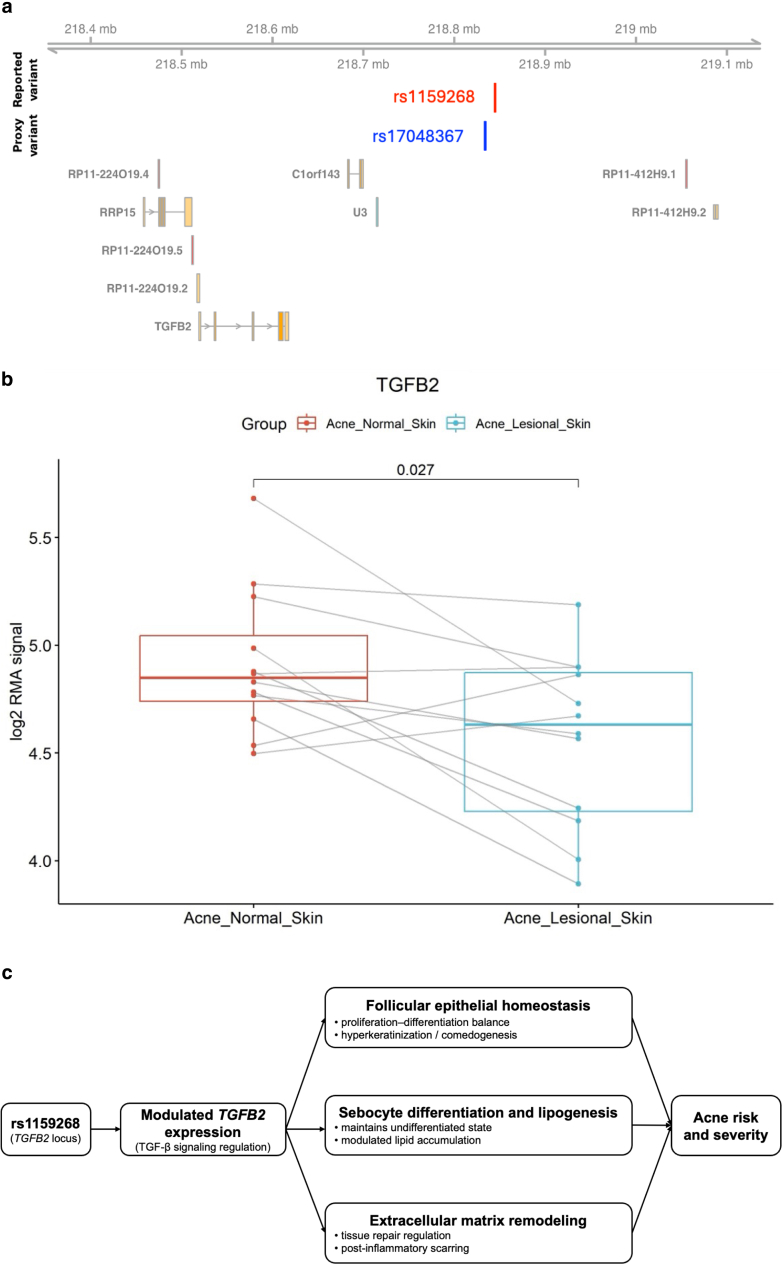
**Functional interpretation of the replicated rs1159268 signal at the *TGFB2* locus.(a)** Genomic context of the reported variant rs1159268 at chromosome 1q41 and the proxy variant rs17048367, which is in perfect linkage disequilibrium with rs1159268 (r^2^ = 1.0) in the 1000 Genomes Han Chinese in Beijing (CHB) reference panel. The reported variant rs1159268 is highlighted in red, and the proxy variant rs17048367 is shown in blue. (**b)***TGFB2* expression levels in paired acne lesional and nonlesional skin samples GSE53795 from Gene Expression Omnibus dataset GSE53795. Each line connects paired samples from the same individual. Statistical significance was assessed using paired testing. (**c)** Proposed mechanistic framework linking rs1159268 to acne risk. The rs1159268 variant may modulate *TGFB2* expression and TGF-β signaling activity. Through effects on follicular epithelial homeostasis, sebocyte differentiation and lipogenesis, and extracellular matrix remodeling, this locus may contribute to acne risk, clinical severity, and postinflammatory scarring.

**Figure 4:**
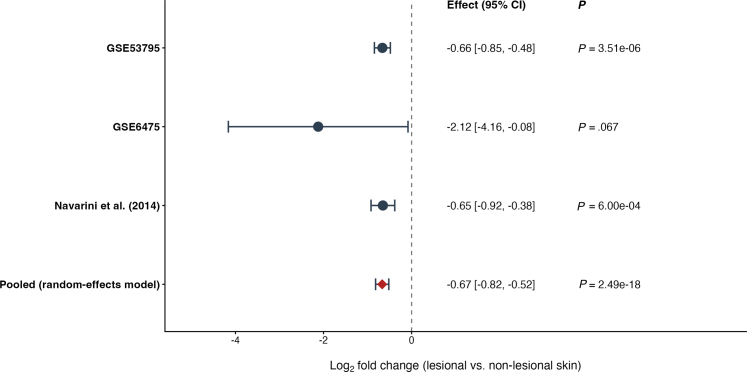
**Meta-analysis of *TGFB2* expression in acne lesional and nonlesional skin.** Shown is a forest plot showing *TGFB2* expression differences between lesional and nonlesional skin across 3 independent cohorts (GSE53795, n = 24; GSE6475, n = 12; Navarini et al, 2014, n = 12). Effect sizes are presented as log_2_ fold change (lesional vs nonlesional skin) with 95% CIs. The diamond represents the pooled estimate from a random-effects model. *TGFB2* expression was lower in lesional skin than in nonlesional skin (pooled log_2_ fold change = −0.67; *P* < .001), with minimal between-study heterogeneity (I^2^ = 0.011%, *P*_heterogeneity_ = .374). CI, confidence interval.

Functional annotation was performed for the lead variant rs1159268 at the 1q41 locus, with *TGFB2* identified as the nearest protein-coding gene (Figure 3a). Transcriptomic analysis revealed a significant downregulation of *TGFB2* in acne lesional skin compared with that in nonlesional skin (Figure 3b). This expression change was further supported by a meta-analysis across 3 independent cohorts (GSE53795, GSE6475, and a clinical dataset (Navarini et al, 2014)), which showed a consistent decrease in *TGFB2* expression (pooled log_2_ fold change = −0.67, *P* < .001) (Figure 4). To investigate the genetic regulation of this pattern, we queried the Genotype-Tissue Expression (GTEx) (version 8) database, which demonstrated that the acne-risk allele [A] of rs1159268 is a significant expression quantitative trait locus (eQTL) for decreased *TGFB2* expression in epithelial-related tissues (normalized enrichment score = −0.30, *P* = 5.31 × 10^−8^) (Supplementary Table S3). This genetically predicted downregulation is highly concordant with the *TGFB2* suppression observed in the lesional skin samples. Furthermore, the proxy rs17048367 (r^2^ = 1.0; CHB) also exhibited a significant eQTL signal for *TGFB2* in cultured fibroblasts (GTEx, version 6; *P* = 1.15 × 10^−5^), reinforcing the regulatory potential of this genomic region. Collectively, these findings support *TGFB2* as the most likely functional candidate gene at the 1q41 locus.

**Figure 5:**
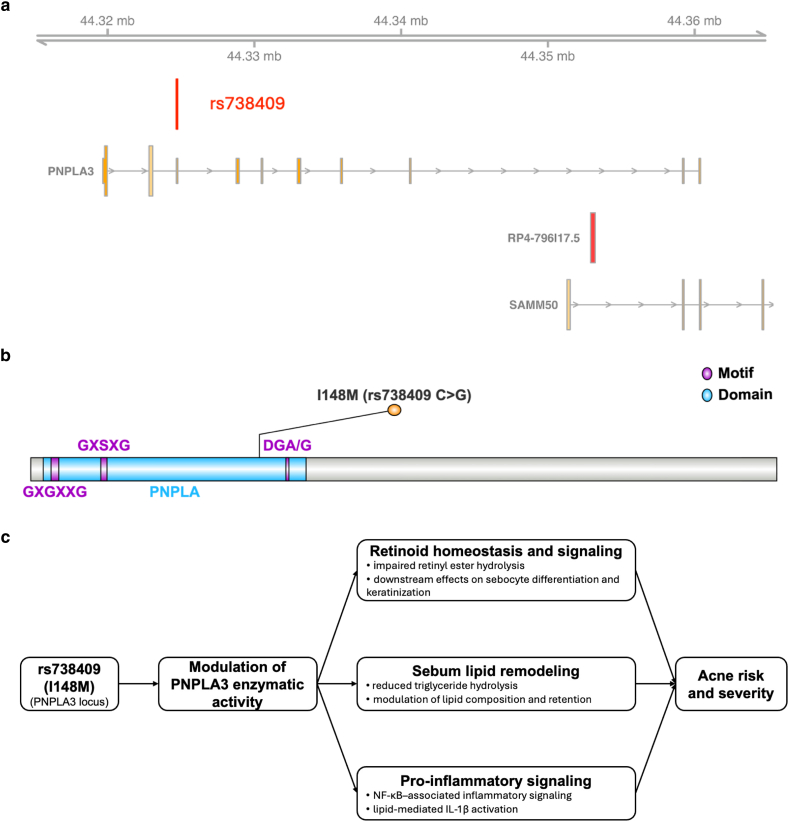
**Functional interpretation of the replicated rs738409 signal at the *PNPLA3* locus.(a)** Genomic context of the replicated variant rs738409 at chromosome 22q13.31. The variant is located within the third exon of *PNPLA3* and results in a missense substitution (I148M). The reported variant rs738409 is highlighted in red. (**b)** Domain organization of PNPLA3 protein showing the I148M missense substitution (orange) within the PNPLA domain (blue). Conserved catalytic motifs are indicated in purple. (**c)** Proposed mechanistic framework linking rs738409 variant to acne risk. The I148M substitution may modulate PNPLA3 enzymatic activity, with downstream effects on retinoid homeostasis, sebum lipid remodeling, and inflammatory signaling. Disruption of retinyl ester metabolism may influence sebocyte differentiation and follicular keratinization, whereas altered triglyceride turnover may modify sebum lipid composition. These changes may also enhance proinflammatory signaling within the pilosebaceous unit, collectively contributing to acne susceptibility and clinical severity. PNPLA, patatin-like phospholipase.

By comparison, rs738409 is located within the exon of *PNPLA3* at 22q13.31 and is annotated as a missense variant (I148M). This substitution is positioned within the patatin-like phospholipase domain (Figure 5a and b), consistent with an impact on protein function. In addition to its missense impact, rs738409 is identified as a significant eQTL for *PNPLA3* in human skin tissues (GTEx, version 8). Specifically, the acne-associated [C] allele correlates with increased *PNPLA3* mRNA expression (*P* < .05) (Supplementary Table S3). This convergent evidence, comprising both a functional protein substitution and a significant regulatory signal, strongly supports *PNPLA3* as the functional candidate gene at the 22q13.31 locus.

### G×E analysis

Given the limited replication of previously reported acne GWAS loci in the SMCGES cohort, the potential modification of genetic associations by environmental exposures was further evaluated. G×E analyses were performed using logistic regression models incorporating variant main additive effects, environmental exposures, and variant × exposure interaction terms, with adjustment for age and sex.

Across the environmental factors assessed, screen-use time showed the most consistent evidence of the significant interaction. On the basis of the variant effect estimated within the interaction model (*P*_variant_), 12 variants met the Bonferroni-corrected significance threshold (*P* = 5.68 × 10^−4^) in the screen-time analysis (Table 3). All 12 variants additionally showed evidence of effect modification by screen-time exposure, with nominally significant variant × screen-time interaction terms (*P*_G×E_ < .05).

**Table 3:** Genetic Variants with Significant Variant × Screen-Time Interaction Effects on Acne Risk

rs ID	Chr:Pos (GRCh37)	Minor/Major Allele	MAF	Main-Effect Model^1^		Interaction Model^2^				Implicated Gene(s)^4^
				*P*_main_	OR_main_ (95% CI)^3^	*P*_variant_	OR_variant_ (95% CI)^3^	*P*_GxE_	OR_GxE_ (95% CI)^3^	
rs1256658	1:219206313	A/G	0.2819	0.0773	0.922 (0.842–1.009)	1.25E-04	0.656 (0.529–0.814)	9.30E-04	1.12 (1.047–1.197)	*LYPLAL1-DT*
rs6698475	1:219633525	A/C	0.3489	0.1757	0.944 (0.867–1.026)	2.08E-05	0.662 (0.547–0.800)	4.54E-05	1.129 (1.065–1.197)	*LAYPLAL-AS1*
rs13013349	2:113600326	T/C	0.4741	0.4763	0.972 (0.897–1.052)	6.34E-05	0.722 (0.616–0.847)	2.73E-05	1.108 (1.056–1.163)	*IL1A*, *IL37, IL36B*, *IL36RN*, *CDK8P2*, *IL1B*, *IL1RN*
rs6842241	4:148400819	A/C	0.2132	0.2907	0.949 (0.861–1.046)	8.54E-05	0.609 (0.476–0.780)	1.56E-04	1.163 (1.075–1.257)	*EDNRA*
rs455660	5:55816888	T/C	0.3880	0.2177	0.950 (0.875–1.031)	2.38E-04	0.712 (0.594–0.853)	5.63E-04	1.103 (1.043–1.166)	*C5orf67*
rs4723979	7:40868141	C/T	0.4763	0.6618	0.982 (0.907–1.064)	3.57E-05	0.715 (0.610–0.839)	5.99E-06	1.118 (1.065–1.173)	*SUGCT*
rs6949092	7:40872428	T/C	0.4756	0.6070	0.979 (0.904–1.061)	6.78E-05	0.723 (0.616–0.848)	1.58E-05	1.112 (1.06–1.167)	*SUGCT*
rs174594	11:61619829	A/C	0.3943	0.3076	0.957 (0.880–1.041)	2.06E-04	0.718 (0.603–0.855)	2.89E-04	1.104 (1.047–1.165)	*FADS2*, *FEN1*, *TMEM258*, *CYB561A3*, *FADS1*, *RAB3IL1*
rs61744384	11:65387378	A/T	0.4806	0.0580	0.925 (0.854–1.003)	3.72E-05	0.715 (0.610–0.839)	2.56E-04	1.093 (1.042–1.147)	*MAP3K11*, *RNASEH2C*, *KRT8P26*, *EIF1AD*, *CTSW*, *SNX32*, *RELA-DT*, *NEAT1*, *OVOL1-AS1*, *PCNX3*, *SIPA1*, *BANF1*, *KAT5*
rs11602680	11:69053932	G/A	0.4869	0.0090	0.897 (0.828–0.973)	2.40E-07	0.655 (0.558–0.769)	1.13E-05	1.113 (1.061–1.167)	*MYEOV*
rs7295080	12:12559234	A/G	0.2599	0.2451	0.947 (0.863–1.038)	3.35E-04	0.657 (0.522–0.826)	5.78E-04	1.134 (1.056–1.219)	*BORCS5*
rs12321	22:29453193	C/G	0.4470	0.3150	0.959 (0.885–1.040)	1.13E-04	0.724 (0.614–0.853)	1.09E-04	1.103 (1.05–1.159)	*ZNRF3*

Abbreviations: CI, confidence interval; Chr, chromosome; dbSNP, database of single-nuclotide variant; eQTL, expression quantitative trait locus; G×E, gene–environment interaction; ID, identification; MAF, minor allele frequency; OR, odds ratio; Pos, position; UTR, untranslated region.

1 The main-effect model corresponded to a logistic regression of acne on single-nucleotide variant, adjusted for age and sex; *P*_main_ and OR_main_ represent the variant main effect from this model.

2 The interaction model included variant genotype, screen-time exposure, and the variant × screen-time interaction term, adjusted for age and sex; *P*_variant_ and OR_variant_ represented the variant main effect, and *P*_G×E_ and OR_G×E_ represent the multiplicative G×E interaction effect. Statistical significance for variant main effects (*P*_variant_) was evaluated using a Bonferroni-corrected threshold (*P* < 5.68 × 10^−4^), whereas variant × environment interaction effects were considered at nominal significance (*P*_G×E_ < .05).

3 ORs and 95% CIs were estimated per the minor allele under an additive genetic model.

4 Implicated genes were assigned on the basis of the following criteria: (i) functional annotation of coding, splice-site, or UTR variants to protein-coding genes using dbSNP; (ii) significant eQTLs in acne-relevant GTEx tissues (skin, subcutaneous adipose, cultured fibroblasts, whole blood); or (iii) otherwise, the nearest protein-coding gene.

**Figure 6:**
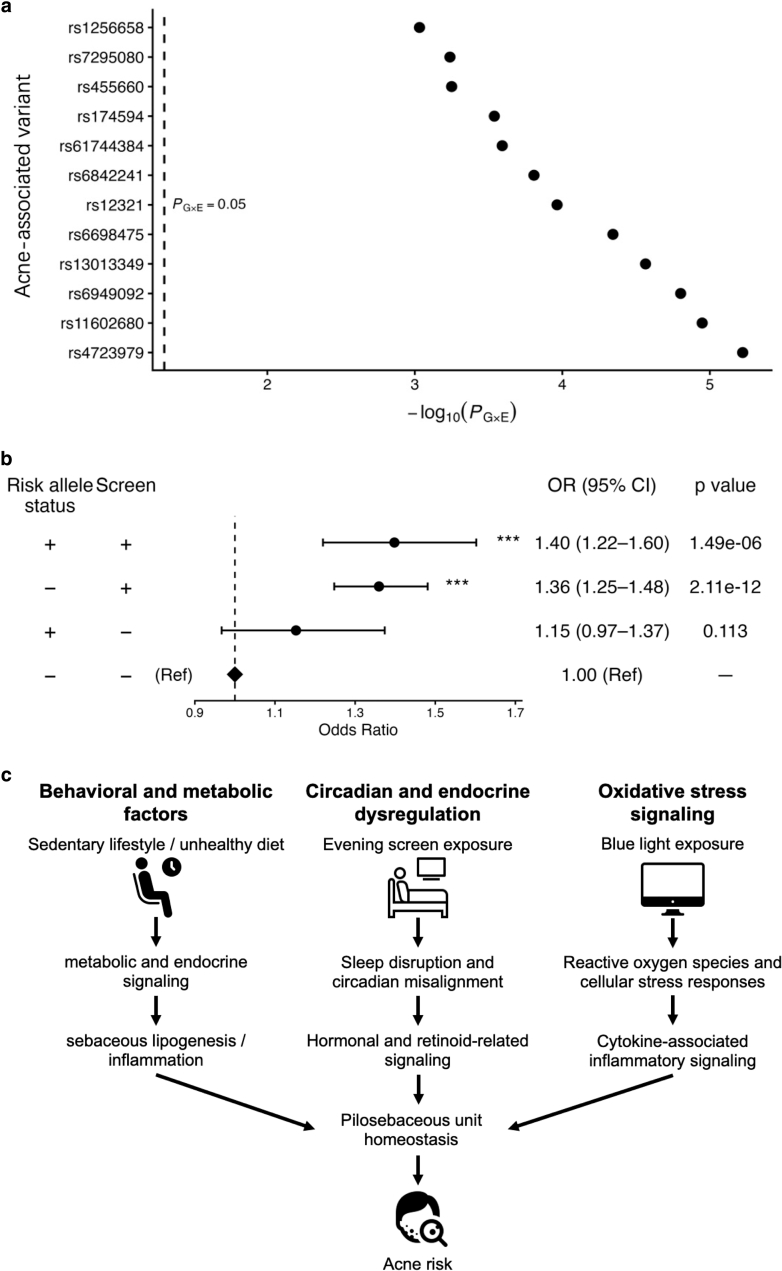
**Visualization of variant × screen-time interactions and proposed mechanisms.(a)** Overview of variant × screen-time interaction signals across the 12 loci identified in the interaction analysis. Signals are shown as −log10(*P*_G×E_) values. *P*_G×E_ represents the *P*-value for the variant × screen-time interaction term estimated from logistic regression models adjusted for age and sex. The dashed horizontal line indicates the nominal interaction significance threshold (*P*_G×E_ = .05). (**b)** Forest plot of the variant showing the strongest interaction signal (rs4723979). ORs and 95% CIs are shown for the four groups defined by risk-allele status and screen-time exposure. “+” represents 2 risk alleles or screen use >3 hour/day, “−” indicates 0–1 risk allele or screen use ≤3 hour/day. ORs and *P*-values are estimated on the basis of the low-risk/low-screen reference group (−/−). Statistical significance is indicated by asterisks (∗*P* < .05, ∗∗*P* < .01, and ∗∗∗*P* < .001). The effect observed in the high-risk/high-screen group (+/+) differs from that expected on the basis of the independent effects of genotype and screen time. Under independent combination, the joint effect would be expected to approximate the product of the individual effects. This deviation is consistent with the significant variant × screen-time interaction. (**c)** Proposed mechanisms through which screen-related exposures may interact with genetic susceptibility to influence acne risk. These include (i) behavioral and metabolic factors related to sedentary lifestyle and associated endocrine signaling, (ii) circadian and endocrine dysregulation linked to sleep disruption and light exposure, and (iii) oxidative stress–related signaling involving cellular stress responses and inflammatory pathways. These processes may converge on pilosebaceous unit homeostasis, thereby modifying the effect of genetic susceptibility on disease manifestation. CI, confidence interval; G×E, gene–environment interaction; OR, odds ratio.

**Figure 7:**
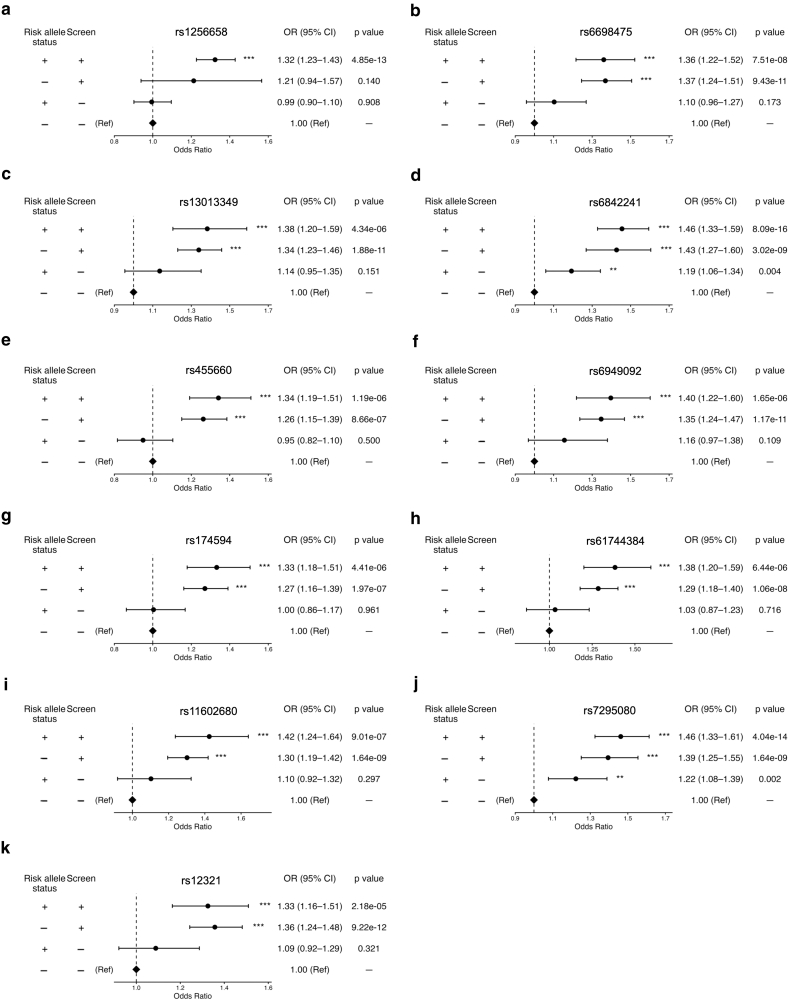
**Forest plots of variant × screen-time interactions across remaining loci.** ORs and 95% CIs are shown for the 4 groups defined by risk-allele status and screen-time exposure. “+” represents risk alleles or screen use >3 hour/day, and “−” indicates 0–1 risk allele or screen use ≤3 hour/day. ORs are estimated relative to the low-risk/low-screen reference group (−/−). Statistical significance is indicated by asterisks (∗*P* < .05, ∗∗*P* < .01, and ∗∗∗*P* < .001). Deviations of the observed joint effect (+/+) from that expected under independent combination are consistent with variant × screen-time interactions.CI, confidence interval; OR, odds ratio.

Figure 6a shows that all 12 loci exhibit nominally significant variant × screen-time interaction signals, with a clear gradient in interaction strength. Among these, rs4723979 displays the strongest interaction signal. To further characterize the joint effects underlying these interactions, stratified ORs were examined across combinations of genetic risk and screen-time exposure (Figures 6b and 7). For rs4723979, the estimated effect in the high-risk/high-screen group (+/+) deviates from that expected under independent combination of genetic risk and screen-time exposure, where the joint effect would be approximated by the product of the stratum-specific effects observed in the high-screen/low-genetic-risk group (−/+) and the high-genetic-risk/low-screen group (+/−). Similar patterns of deviation were observed across the remaining loci, although the magnitude of deviation varied across variants (Figure 6).

## Discussion

### *TGFB2* at 1q41: implicating TGF-β signaling in acne susceptibility

The replicated association at the 1q41 locus, indexed by rs1159268, aligns with previous GWAS findings implicating this region in acne susceptibility (Mitchell et al, 2022; Navarini et al, 2014; Petridis et al, 2018). Our functional annotation identifies *TGFB2* as the primary candidate gene at this locus. Supporting this, GTEx (version 8) analysis shows that the acne-risk allele [A] of rs1159268 is a significant eQTL for decreased *TGFB2* expression in epithelial tissues. This genetically predicted downregulation is highly concordant with the robust *TGFB2* suppression observed in our cross-cohort meta-analysis (Figures 3b and 4). Although this eQTL signal was not directly from skin tissues, previous studies have shown that a substantial proportion of regulatory effects are shared across biologically distinct tissues owing to conserved regulatory mechanisms (Ip et al, 2018). The regulatory potential of this locus is further supported by the proxy variant rs17048367 (r^2^ = 1.0; CHB), which demonstrates significant eQTL effects in fibroblasts (GTEx, version 6) (Supplementary Table S3) and overlaps with putative enhancer elements in epidermal keratinocytes (Supplementary Table S4). Although the exact molecular pathways remain to be fully characterized, our findings suggest that genetic variation at 1q41 may influence acne susceptibility by modulating *TGFB2* expression levels.

*TGFB2* encodes TGF-β2, a multifunctional cytokine of the TGF-β superfamily, which plays a central role in epithelial homeostasis and tissue remodeling. TGF-β signaling regulates keratinocyte proliferation and differentiation and is involved in hair follicle development and cycling, processes that are essential for maintaining pilosebaceous unit integrity (Deng et al, 2024; Inoue et al, 2009). Accordingly, TGF-β signaling may influence acne risk by modulating the balance between proliferation and differentiation in the follicular epithelium, thereby contributing to follicular hyperkeratinization and early comedone development, as summarized in Figure 3c. This is consistent with evidence from recent large-scale genetic studies, which indicate that acne susceptibility is largely driven by variation affecting pilosebaceous unit structure and tissue homeostasis (Mitchell et al, 2022; Petridis et al, 2018; Van Steensel, 2025). In addition to these roles, TGF-β signaling also regulates sebocyte differentiation and lipogenesis, with pathway activation maintaining sebocytes in a relatively undifferentiated state and suppressing lipid accumulation (McNairn et al, 2013). Furthermore, given the central role of TGF-β signaling in extracellular matrix remodeling and fibrosis, it may also influence the risk of postinflammatory scarring after severe acne lesions (Xu et al, 2024).

### *PNPLA3* at 22q13: implicating retinoid metabolism in acne susceptibility

The replicated signal at the 22q13 locus localizes within *PNPLA3* coding region. The rs738409 variant results in an isoleucine-to-methionine substitution (I148M) and has been extensively characterized as a functional allele that modulates PNPLA3 activity at the lipid droplet surface (He et al, 2010; Pirazzi et al, 2014; Wang et al, 2025, 2019). The regulatory evidence at this locus provides an additional layer of functional insight. Our integration of GTEx (version 8) data identifies rs738409 as a significant eQTL in human skin, where the risk allele correlates with increased *PNPLA3* expression. This dual functional and regulatory impact strongly reinforces *PNPLA3* as the most potential candidate gene of the association signal at 22q13. Notably, although PNPLA3 is predominantly recognized for its role in hepatic lipid metabolism, systemic transcriptomic profiling (GTEx, version 8) and previous studies (Bruschi et al, 2017) confirm its substantial expression in human skin, suggesting that similar lipid-regulatory mechanisms may contribute to acne pathogenesis.

*PNPLA3* encodes a lipid droplet–associated enzyme involved in triglyceride and retinyl ester metabolism. The acne-associated rs738409 (I148M) variant may therefore provide a mechanistic link between lipid droplet dynamics and pilosebaceous unit homeostasis, as illustrated in Figure 5c. In addition to its role in triglyceride remodeling, PNPLA3 has retinyl-palmitate hydrolase activity and contributes to intracellular retinoid homeostasis (Kovarova et al, 2015; Pirazzi et al, 2014). Given the established role of retinoid signaling in pilosebaceous unit biology, including sebocyte differentiation and follicular keratinization, variation in retinoid availability associated with the I148M variant may influence pathways relevant to comedone development (Durgin et al, 2026; Everts, 2012). PNPLA3 also participates in triglyceride turnover and polyunsaturated fatty acid mobilization, raising the possibility that rs738409 may contribute to changes in sebum lipid composition relevant to acne biology (Del Rosso and Kircik, 2024; Johnson et al, 2024; Luukkonen et al, 2019; Okoro et al, 2021). PNPLA3 has also been linked to enhanced inflammatory signaling in other disease models. Such changes in lipid composition may promote proinflammatory signaling within the pilosebaceous unit and thereby contribute to lesion progression (Jung et al, 2021; Yuan et al, 2020).

### Screen time as a contextual modifier of genetic effects on acne

Our interaction analyses suggest that screen-use duration modifies the association between acne risk alleles and disease presentation at a subset of loci, with evidence of locus-specific heterogeneity rather than a uniform interaction pattern. Functional annotation of the screen-time–prioritized variants revealed enrichment of Gene Ontology terms related to inflammatory signaling, lipid metabolism, and cellular responses to oxidative stress (Supplementary Table S5). These enriched processes are relevant to acne biology, but they are also pathways that are sensitive to metabolic state and environmental exposures (Grafanaki et al, 2026; Van Steensel, 2025). Recent studies further indicate that oxidative stress, lipid imbalance, and cytokine signaling are closely linked to pilosebaceous unit function and can influence keratinocyte differentiation, sebaceous activity, and local tissue homeostasis (Durgin et al, 2026; Li et al, 2025). In this context, our enrichment results suggest that screen-associated exposures may influence the biological environment of the pilosebaceous unit and thereby modify the effect of genetic susceptibility on disease manifestation.

Building on these observations, we propose a conceptual framework in which screen-associated exposures may influence acne risk through 3 possible pathways (Figure 6c): (i) behavioral and metabolic factors linked to sedentary lifestyle and diet, (ii) circadian and endocrine dysregulation, and (iii) oxidative stress–related signaling.

Among the potential mechanisms, the most plausible is the clustering of behaviors associated with prolonged screen use. Epidemiological studies consistently show that higher screen time is associated with sedentary behavior; reduced physical activity; and less favorable dietary patterns, including higher intake of energy-dense or high-glycemic foods (Alnaqbi et al, 2025; Bejarano et al, 2021; Rocka et al, 2022). These factors are closely linked to endocrine and metabolic pathways relevant to acne, particularly insulin-like growth factor 1 signaling, sebaceous activity, and lipogenesis (Bungau et al, 2023; Mosca et al, 2025). Although individual measures of physical activity and diet were not significantly associated with acne in our cohort (Table 1), such variables may not fully capture the nuances of metabolic exposures. Screen time may therefore serve as a composite proxy for clustered lifestyle behaviors, reflecting underlying metabolic and endocrine contexts that are difficult to quantify through current survey measures.

Higher screen exposure, especially in the evening, has also been associated with sleep disruption and circadian misalignment, and experimental studies have shown that light-emitting devices can suppress melatonin secretion and delay circadian phase (Chang et al, 2015a; Chinoy et al, 2018). Such perturbations may influence hormonal and retinoid-related signaling within the skin, processes that are closely tied to keratinocyte differentiation and pilosebaceous unit homeostasis (Durgin et al, 2026; Sadur et al, 2025; Samaniego et al, 2025). By contrast, although blue light can induce oxidative stress and inflammatory signaling in in vitro skin models, direct evidence for a substantial in vivo effect on acne is still limited (Montero et al, 2023; Werner et al, 2025). Overall, these findings support a model in which screen time acts more as a contextual exposure that may interact with genetic susceptibility to acne.

### Conclusions and future directions

In summary, this study replicated previously reported acne-associated variants in the SMCGES cohort and linked them to genes involved in pilosebaceous unit biology. Notably, the replicated signals were derived from European-ancestry studies, whereas certain previously reported Han Chinese loci did not reach significance, likely reflecting population-specific genetic architecture and linkage disequilibrium patterns. G×E analyses further identified 12 variants whose effects were modified by screen-time exposure, supporting a model in which screen-associated exposures may shape the biological context of acne genetic susceptibility.

Several limitations should be noted. Screen-time exposure was self-reported, which may introduce recall and reporting bias. The functional effects of multiple variants require experimental validation. Analyses were restricted to individuals of Chinese ancestry, which may limit the applicability to other populations. In addition, potential confounders, such as psychosocial factors, were not fully accounted for owing to data scarcity across cohorts, requiring the cautious interpretation of the GxE interactions. Future studies should incorporate objective measures of digital exposure and repeated exposure assessment over time. Replication in cohorts with diverse ancestral backgrounds is required to evaluate generalizability. Functional and experimental studies are needed to define the molecular mechanisms underlying the identified loci and G×Es. Together, these results show that both genetic and environmental factors contribute to acne susceptibility and provide a foundation for future mechanistic and translational research.

## Materials and Methods

### Literature search and acne-associated variant selection for replication

A systematic literature search was conducted in Web of Science and PubMed to identify previously published acne GWAS. Search terms included “acne” OR “acne vulgaris” combined with “genome-wide association” OR “GWAS.” Peer-reviewed original studies reporting genome-wide or suggestive associations with (i) acne presence versus absence or (ii) clinically graded acne severity were eligible. All reported variants meeting the prespecified significance threshold (*P* < 1 × 10^−5^) were collated, and duplicated signals across studies were merged into unique loci; when the same locus was reported in multiple cohorts, the most significant index variant from the original publication was retained.

### Study design, ethics, and participants

Participants were drawn from the SMCGES dataset, an ongoing epidemiology collection conducted across 3 universities (National University of Singapore, Universiti Tunku Abdul Rahman, and Sunway University) over multiple recruitment waves (Huang et al, 2025). For the present analysis, participants were restricted to individuals of Chinese ancestry to minimize potential confounding owing to population stratification. Written informed consent was obtained from all participants prior to enrollment. The study was conducted in accordance with the Declaration of Helsinki and approved by the relevant ethics committees in Singapore and Malaysia, including approvals from the National University of Singapore Institutional Review Board (Heng et al, 2022) and the ethics approvals from Universiti Tunku Abdul Rahman and Sunway University in Malaysia (Say et al, 2021). Demographic information and environmental exposure variables were collected using a validated investigator-administered questionnaire adapted from s the standardized International Study of Asthma and Allergies in Childhood protocol (Huang et al, 2025).

### Disease definition

Acne status was defined using harmonized criteria previously adopted in SMCGES epidemiological analyses (Huang et al, 2025). Participants were classified as acne cases if they answered “yes” to either “Have you ever visited a doctor for your acne condition?” or “Do you have scars (keloids) left by acne/boils?” and/or met the case definition on the basis of on-site dermatological assessment as described in the SMCGES acne phenotype protocol. Participants were classified as acne controls if they answered “no” to both questions and did not meet the assessment-based case definition.

### Genotyping, imputation, and quality control

Genotyping was performed using 5 different genotyping array platforms: Infinium OmniZhongHua-8 (version 1.3/version 1.4), Illumina HumanHap550K, Infinium Omni2.5-Exome, Infinium Global Screening Array, and Infinium Asian Screening Array. Detailed platform specifications, including marker density and sample distributions, are provided in Supplementary Table S6. Standard sample- and variant-level quality control was performed independently for each dataset to ensure genotyping integrity. Phasing and imputation were subsequently conducted using IMPUTE2 (Howie et al, 2009) with the CHB reference panel (Fairley et al, 2019). Postimputation data quality was assessed using established metrics, and downstream association tests were restricted to directly genotyped variants or high-quality proxies (r^2^ ≥ 0.8).

### Association testing and multiple-testing control

Primary analyses were performed using logistic regression under an additive model. To account for potential population stratification, models were adjusted for age, sex, and the top 10 principal components. Association testing was conducted using PLINK, version 2.0 (Chang et al, 2015b). For each variant, ORs, 95% confidence intervals, and Wald test *P*-values were estimated. To account for multiple testing across the set of candidate variants evaluated for replication, statistical significance was determined using a Bonferroni-corrected threshold on the basis of the total number of variants tested.

### G×E analysis

To evaluate whether environmental exposures modify genetic associations with acne, G×E analyses were conducted. Environmental factors were first assessed for their independent associations with acne susceptibility. Variables demonstrating robust and consistent phenotypic correlations across all exposure levels were prioritized for the following GxE interaction analysis. Interaction effects were modeled using multivariable logistic regression, adjusting for age and sex. For transparency, interaction analyses for all other environmental factors were conducted in parallel and reported as exploratory data in Supplementary Table S2. All the interaction analyses were implemented using PLINK (version 2.0) (Chang et al, 2015b) and R (version 4.5.1) (Team RC, 2025).

Interaction effects were assessed on the multiplicative scale, and statistical evidence for effect modification was evaluated using the Wald test for the interaction term. Variants showing statistically significant evidence of interaction with environmental exposures according to the study’s prespecified criteria were prioritized for downstream functional mapping and pathway-level analyses.

### Functional annotation

Public genome annotation resources were used to characterize the genomic context of analyzed variants. Genomic position, gene proximity, and predicted functional consequences were obtained using Ensembl (Dyer et al, 2024) and the UCSC Genome Browser (Perez et al, 2025). Regional genomic context was summarized using a standardized window extending 500-kb upstream and downstream of each locus. Visualization of regional genomic features was performed in R using the Gviz package (Hahne and Ivanek, 2016).

### Regulatory and transcriptomic evidence

eQTL information was primarily obtained from GTEx, version 8 (GTEx Consortium, 2020), with GTEx (version 6) utilized as a supplementary resource to ensure a comprehensive functional characterization. Regulatory associations were reviewed across acne-related tissues, including skin, adipose, fibroblasts, and blood. Significance thresholds and false discovery rate control were applied as reported by the respective GTEx releases. Functional annotations of candidate variants were further assessed using Haploreg (version 4.2) (Ward and Kellis, 2016) to examine regulatory features, including chromatin states, transcription factor binding, and conservation across tissues. Public transcriptomic data of acne lesional and nonlesional skin were retrieved from the Gene Expression Omnibus (Barrett et al, 2012) under accession GSE53795 (Kelhälä et al, 2014). Processed and normalized expression matrices provided by the original study were used to compare paired samples, following the procedures described by the dataset authors.

### Pathway enrichment and protein–protein interaction analysis

Functional enrichment analysis was conducted using the STRING platform (Szklarczyk et al, 2024). The foreground gene set comprised protein-coding genes mapped from the 12 G×E significant variants (Table 3), with all annotated human protein-coding genes utilized as the background universe. Over-representation analysis was performed for Gene Ontology biological process terms, with significance thresholds maintained as reported by STRING (Szklarczyk et al, 2024). Multiple testing corrections were applied using the Benjamini–Hochberg method, with a false discovery rate <0.05 considered statistically significant.

## Ethics Statement

This study was conducted in accordance with the principles of the Declaration of Helsinki. Ethical approval for participant recruitment and study procedures was obtained from the following institutional review bodies: the National University of Singapore Institutional Review Board (approval numbers 07–023, 09–256, 10–445, 13–075, B-10-343, and H-18-036); the Scientific and Ethical Review Committee of Universiti Tunku Abdul Rahman, Malaysia (approval number U/SERC/03/2016); and the Research Ethics Committee of Sunway University, Malaysia (approval number SUREC 2019/029). Written informed consent was obtained from all participants prior to enrolment. For participants aged <21 years, additional written informed consent was obtained from a parent, legal guardian, or next of kin.

## Data Availability Statement

The summary-level statistical data generated in this study and underlying the reported analyses have been deposited in Mendeley Data and are available at https://doi.org/10.17632/c39pp47nwy.1. Further information is available from the corresponding author (FTC) upon reasonable request.

## ORCIDs

Lingshu Liu: http://orcid.org/0009-0008-5673-9388

Yang Yie Sio: http://orcid.org/0000-0002-3549-1706

Dingyu Cen: http://orcid.org/0009-0004-9608-564X

Jia Yi Karen Wong: http://orcid.org/0009-0006-2800-4451

Xiangran Chen: http://orcid.org/0009-0000-5496-2940

Yee-How Say: http://orcid.org/0000-0003-2363-5239

Kavita Reginald: http://orcid.org/0000-0003-1530-5934

Fook Tim Chew: http://orcid.org/0000-0003-1337-5146

## Conflict of Interest

FTC reports grants from the National University of Singapore, Singapore Ministry of Education Academic Research Fund, Singapore Immunology Network, National Medical Research Council (Singapore), Biomedical Research Council (Singapore), National Research Foundation (Singapore), Singapore Food Agency, Singapore’s Economic Development Board, and the Agency for Science Technology and Research (Singapore), during the conduct of the study, and consulting fees from Sime Darby Technology Centre, First Resources, Genting Plantation, Olam International, Musim Mas, and Syngenta Crop Protection, outside the submitted work. The remaining authors state no conflict of interest.

## Footnotes

Supplementary material is linked to the online version of the paper at www.jidonline.org, and at 10.1016/j.xjidi.2026.100490.
